# Age as a Predictor of Serum Tumor Necrosis Factor Antagonist Drug and Antidrug Antibody Concentrations in Inflammatory Bowel Disease—A Nationwide Cohort Study

**DOI:** 10.3390/jcm14041057

**Published:** 2025-02-07

**Authors:** Mohammad Shehab, Fatema Alrashed

**Affiliations:** 1 Department of Internal Medicine, Mubarak Al-Kabeer University Hospital, Aljabreyah 47060, Kuwait; dr_mshehab@hotmail.com; 2 Department of Translational Medicine, Dasman Diabetes Institute, Dasman 15462, Kuwait; 3 Department of Pharmacy Practice, College of Pharmacy, Kuwait University, Aljabreyah 13110, Kuwait

**Keywords:** IBD, TDM, anti-TNF agent, immunogenicity

## Abstract

**Background/Objectives**: Tumor necrosis factor antagonists (anti-TNFs) have been shown to be an effective treatment for inflammatory bowel disease (IBD). Several factors are associated with anti-TNF treatment failure. This study aims to explore the impact of age on serum concentrations of anti-TNF drugs and antidrug antibodies (ADAbs). **Methods**: We retrospectively reviewed patients’ charts from July 2018 until September 2024 across seven medical centers. Patients with an established diagnosis of IBD receiving infliximab or adalimumab were included. The primary outcome of this study was the effect of age on the anti-TNFs serum drug concentration and ADAb levels. Linear regression was performed to explore the relationship between age and serum anti-TNF drug and ADAb levels. **Results**: 1093 patients were included in our cohort. In patients receiving infliximab, there was a significant association between older age and increasing ADAbs levels (*p* = 0.036), whereas in patients treated with adalimumab, there was no significant relationship between older age and ADAb levels (*p* = 0.771). There was no significant relationship between age and adalimumab serum concentration (*p* = 0.54). When stratified by age, patients taking infliximab who were >30 years of age developed more ADAbs compared to those aged ≤30 (*p* = 0.003). **Conclusions**: Patients older than 30 years of age receiving infliximab monotherapy have higher ADAbs and lower serum drug concentrations than younger patients. There was no statistically significant difference in ADAbs and serum drug concentrations among patients receiving infliximab combination therapy or adalimumab monotherapy.

## 1. Introduction

Inflammatory bowel disease (IBD), including ulcerative colitis (UC) and Crohn’s disease (CD), is a chronic disease that is characterized by inflammation in gastrointestinal tract. Treatment of IBD is dependent on the severity of the disease, which can range from mild to severe [1].

The tumor necrosis factor inhibitors (anti-TNFs) include infliximab and adalimumab. Ani-TNFs are effective treatments for patients with moderate to severe IBD, in both CD and UC. Favorable outcomes such as clinical remission, mucosal healing, and endoscopic remission have been reported following successful treatment with anti-TNFs [2,3].

However, treatment failure after receipt of these agents has been a major drawback, with multiple etiologies being implicated [4,5]. Treatment failures can be primary, occurring during the induction phase, or secondary, occurring later during the maintenance phase of treatment.

Disease-, patient-, and drug-specific predictors have been implicated as reasons for anti-TNF treatment failure. The effect of serum drug and antidrug antibody (ADAb) concentrations has been explored in the literature [6,7,8]. Studies have reported the association between low serum drug concentrations and anti-TNF treatment failure, which is partly mediated by immunogenicity. Furthermore, a study showed that ADAbs against adalimumab and infliximab were significantly higher in the pediatric population than in the adult population [9]. In addition, a significant association between serum levels of anti–tumor necrosis factor agents and the level of mucosal healing has been reported [10].

Genetic factors such as HLADQA1*05 have been shown to be associated with anti-TNF failure [11,12], and studies have shown a high frequency of HLA-DQ genotypes such as (HLA)-DQ2.5 protein (encoded by HLA-DQA1*05) in some Middle Eastern populations [13,14]. In addition, heterogenicity of diseases such as perianal fistulizing Crohn’s is another reason for treatment failure. Shehab et al. cited gender as one of the factors affecting serum anti-TNFs and ADAbs concentration [15]. Another study reported a reduction in infliximab serum drug concentration over time in patients with IBD [16]. The use of age as a predictor of serum anti-TNFs and ADAbs concentration pattern has not been extensively explored in the literature. Furthermore, early identification of factors that might predict treatment failure in patients with IBD allows for dose optimization and the use of other strategies to maximize the effectiveness of anti-TNF therapy. Thus, this study aims to investigate the effect of age on serum anti-TNFs and ADAbs concentrations.

## 2. Materials and Methods

### 2.1. Study Design

This was a nationwide, multicenter retrospective cohort study. The chart review was performed by assessing patients’ charts from July 2018 until September 2024. Patients’ electronic medical records were reviewed, and data were collected from seven different hospitals in Kuwait. This study was performed and reported in accordance with Strengthening the Reporting of Observational Studies in Epidemiology (STROBE) guidelines [17]. Ethical approval for this study was obtained by the standing committee for coordination of health and medical research at the ministry of health of Kuwait (IRB 2022/1410) and as per the updated guidelines of the Declaration of Helsinki (64th WMA General Assembly, Fortaleza, Brazil, October 2013) and of the US Federal Policy for the Protection of Human Subjects. Patients’ consent was waived. Both adult and pediatric patients were included.

The inclusion criteria were as follows: (1) patients with an established diagnosis of IBD, (2) receiving adalimumab or infliximab, (3) with available results for an antidrug antibody and/or serum drug trough concentrations reactively or proactively, and (4) receiving a regular standard dose of anti-TNF therapy for at least 6 weeks at the time of outcome measurement. Regular standard dosing of infliximab was 5 mg/kg at 0, 2, 6, then every 8 weeks, and the adalimumab dose was 160 mg at week 0, 80 mg at week 2, then 40 mg every other week.

IBD diagnosis was made according to the international classification of diseases (ICD-11 version: 2024). Patients were considered to have IBD when they had ICD-11 DD70 corresponding to Crohn’s disease (CD) and ICD-DD71 corresponding to ulcerative colitis (UC) [18].

Both serum drug and ADAbs concentrations were measured at the same time. Reactive therapeutic drug monitoring (TDM) testing for adalimumab or infliximab was performed at trough levels before the next scheduled dose. Proactive TDM testing for adalimumab was performed at week 6, whereas TDM for infliximab was performed at week 14. Any random testing, beyond the above scheduled times, was excluded. Patients who were not taking their medicine at regular intervals were excluded. Any patient who received an oral or intravenous formulation of methylprednisolone, budesonide, prednisone/prednisolone, or any other corticosteroid agent within two weeks of measurement were excluded. Finally, any patients with a past medical history of other autoimmune diseases, or those who were taking immunosuppressants for other medical conditions were excluded.

Demographics characteristics of participants were collected retrospectively at the time of serum drug/antibody concentration measurements. These included age, sex, ethnicity, body mass index (BMI), and type of IBD (CD or UC).

### 2.2. Outcomes and Definitions

The primary outcome of this study was the effect of age on infliximab and adalimumab serum drug concentration and ADAb levels.

Additionally, sub-analysis was conducted to ease the impact of anti-TNF combination therapy (adalimumab or infliximab plus an immunomodulator). Moreover, patients taking only infliximab or adalimumab were categorized to be on monotherapy, while patients who received an immunomodulator (such as methotrexate, azathioprine, or 6-mercaptopurine) simultaneously with adalimumab or infliximab were classified as receiving combination therapy. Patients were required to undergo combination therapy for at least 6 months to be classified as part of the combination therapy group.

Collected samples of ADAb and serum drug concentration were measured at one central immunology laboratory. To determine drug tolerance, a homogeneous mobility shift assay (HMSA, Prometheus Laboratories Inc., San Diego, CA, USA) was used for all study participants. The assays were performed by incubating the TNF-488/IC with serum samples or calibration standards to reach equilibrium. As in the ATI-HMSA method, the reaction mixtures were then filtered and analyzed by the SE-HPLC system. Serum drug concentrations/ADAb levels were collected only before the next scheduled dose, i.e., at trough levels. Trough serum drug and ADAb concentrations were performed either reactively, or proactively to optimize the therapy as per each physician’s clinical judgment and practice.

With regard to levels cutoffs, a serum trough level of ≥5, and ≥7.5 µg/mL was considered therapeutic for infliximab and adalimumab, respectively. ADAbs were considered to be detectable at levels >5 AU/mL for infliximab or >10 AU/mL for adalimumab.

### 2.3. Statistical Analysis

Analyses were conducted using SAS 9.4 (SAS Institute, Cary, NC, USA). The relationship between ADAbs and serum drug concentration according to anti-TNF drug type (infliximab and adalimumab) was evaluated using linear regression and Spearman correlation coefficients (r). Regression was used to determine the appropriate age cutoff point, and a sub-analysis was performed according to the detected age cutoff point. Descriptive analyses were conducted to calculate the interquartile range (IQR) and proportions of categorical variables in the total study sample. The statistical significance level was set to α = 0.05 for all association analyses.

In the total study sample analysis, the effect of patient age on ADAb and serum drug concentrations of both adalimumab and infliximab was assessed. ADAbs and serum drug concentrations were analyzed as continuous variables while applying log-transformation to account for skewness in the data and maximize the value of the continuous measurement. Given that we regressed log10-transformed ADAbs and serum drug concentrations, taking the antilog of the linear regression coefficients (β) yielded an adjusted ratio of geometric means (aRoGM), not the difference between geometric means. Hence, the related 95% confidence intervals (CIs) represent limits for RoGM with a null value of “1.”

Associations were adjusted for sex and active inflammation. Active inflammation was defined by any of the following factors: (1) a partial Mayo score of >2 with individual subscores for ulcerative colitis and a Harvey–Bradshaw Index (HBI) score >5 for Crohn’s disease, (2) stool fecal calprotectin (Fcal) of more than 250 µg/g, (3) C-reactive protein (CRP) levels above the upper limit of normal (>10 mg/L), or (4) current corticosteroids steroids therapy within 14 days of serum drug/ADAb concentration measurements. Additional analyses were conducted to determine whether other co-variates can affect the drug or ADAb serum levels. The effect of BMI, albumin, and smoking status were assessed by using the Wilcoxon rank sum test. BMI was categorized as obese (≥30 kg/m^2^) or other (<30 kg/m^2^). Albumin was categorized as normal (≥40 g/L) or abnormal (<40 g/L). Finally, smoking status was categorized using the terms smoker or non-smoker.

## 3. Results

**Figure 1:**
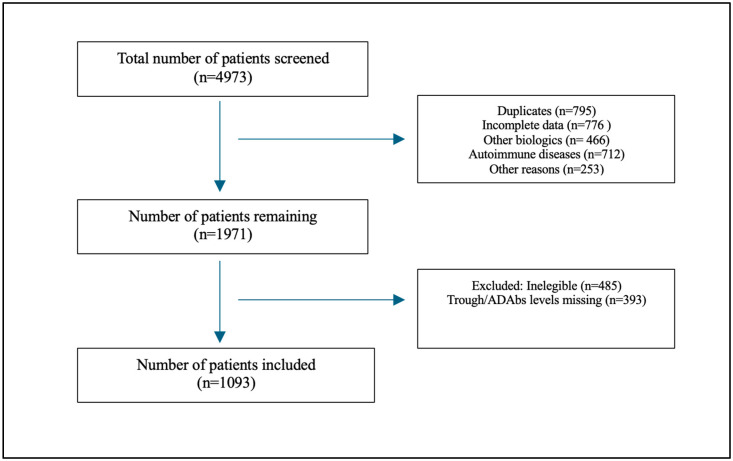
Flow chart showing patient recruitment process.

From July 2018 to September 2024, 4973 patients were screened (see Figure 1 for details on the enrollment process). After the study’s inclusion and exclusion criteria were applied, 1093 patients were included, of which 664 (60.7%) were ≤30 and 429 (39.3%) were >30. The median age was 26 years old (IQR: 19.0–36.0). The youngest patient was 5 years old (two patients), and the oldest patient was 81 years of age (three patients). Males comprised 574 (52.2%) of the total cohort. The median body mass index (BMI) was 23.1 (22.0–24.2) and the majority were Middle Eastern [1007 (92)]. Among the total cohort of patients, 607 (55.5) patients had Crohn’s disease (CD) and 486 (44.5) patients had ulcerative colitis (UC). A total of 125 patients (11%) had active inflammation, while the majority [965 (88%)] did not have active inflammation. The mean level of C-reactive protein was 7.8 mg/L; the mean albumin level was 38.9 g/L; and fecal calprotectin mean level was 130.4 µg/g (see Table 1 for details on the demographic characteristics).

**Figure 2:**
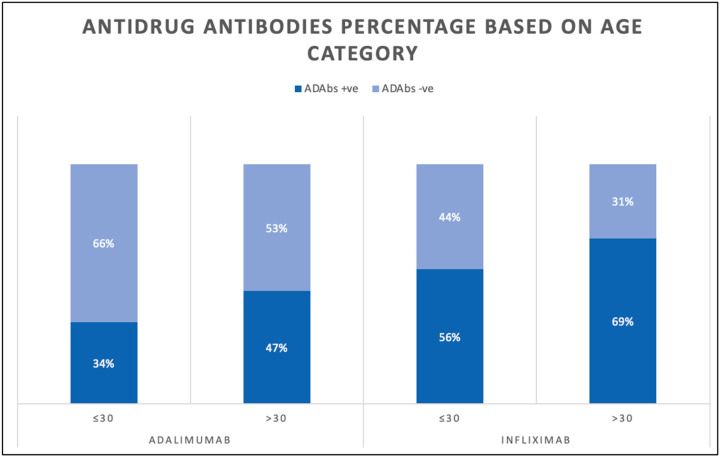
Bar chart showing percentage of antidrug antibodies levels in patients receiving infliximab and adalimumab.

A total of 461 (42.2%) patients were taking infliximab and 632 (57.8%) patients were taking adalimumab. The number of patients receiving combination therapy was 359. Additionally, 147 (40.9%) were prescribed infliximab combination therapy and 212 (59.1%) were prescribed adalimumab combination therapy. The median infliximab therapy duration in years was 4.2 (4.0–4.2), whereas the median adalimumab therapy duration in years was 4.6 (4.0–4.6). The geometric mean (95% CI) of the infliximab serum concentration was 3.3 (2.7–4.1) and the geometric mean (95% CI) of the adalimumab serum concentration was 8.4 µg/mL (7.4–9.5). With regard to antidrug antibodies (ADAbs), the geometric mean (95% CI) of anti-infliximab drug antibody serum levels was 29.0 AU/mL (24.9–33.8), while the geometric mean (95% CI) of anti-adalimumab antibody serum levels was 12.2 AU/mL (10.9–13.7). In patients receiving infliximab, 221 patients (48%) had serum levels of >5 µg/mL, while 277 (60%) had detectable ADAbs. In patients receiving adalimumab, 450 patients (71%) had a serum level of >7.5 µg/mL and 251 (39.9%) had detectable ADAbs. Based on the age category, 175 (56%) patients aged ≤30 and 102 (69%) patients aged >30 had positive infliximab ADAbs. In patients receiving adalimumab, 119 (34%) patients aged ≤30 and 132 (47%) patients aged >30 had positive ADAbs (Figure 2).

### 3.1. Main Outcome

**Figure 3:**
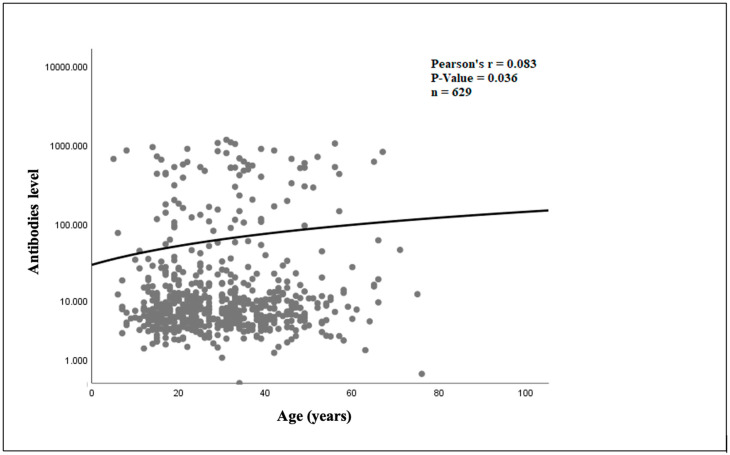
Relationship between age and infliximab antibody level.

**Figure 4:**
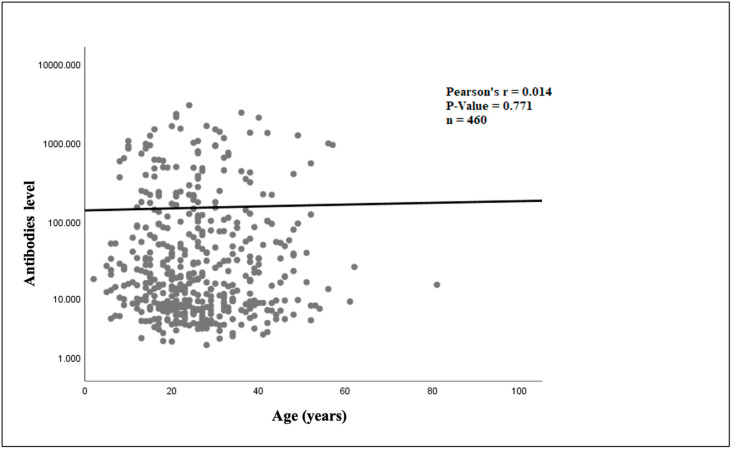
Relationship between age and adalimumab antibody level.

The linear regression analysis showed that in the infliximab cohort (Figure 3), there was a statistically significant relationship between older age and increasing ADAbs levels (*p* = 0.036). In the adalimumab cohort (Figure 4), there was no statistically significant association between older age and ADAbs levels (*p* = 0.771).

**Figure 5:**
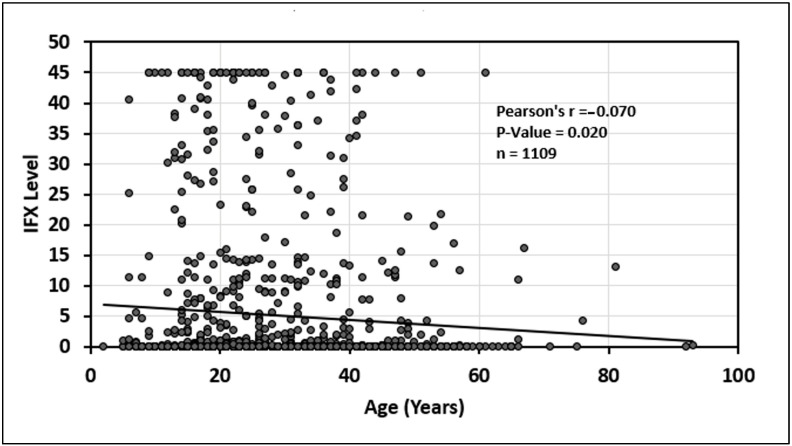
Relationship between age and infliximab (IFX) drug level.

**Figure 6:**
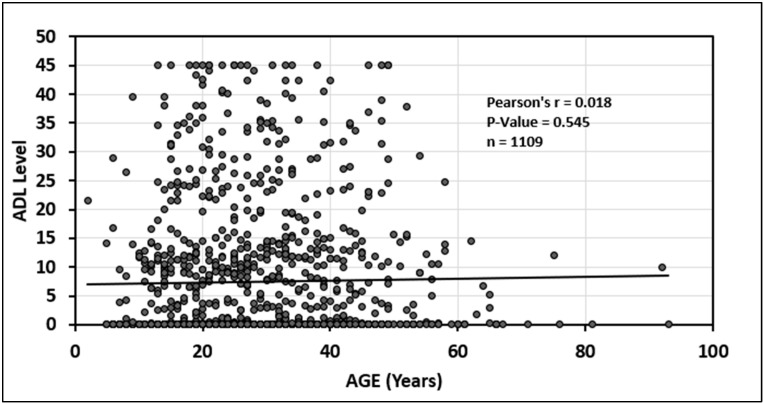
Relationship between age and adalimumab (ADA) drug level.

In addition, there was a statistically significant relationship between older age and decreasing infliximab serum trough levels (*p* = 0.020) see Figure 5. Finally, there was no statistically significant association between age and adalimumab serum trough level (*p* = 0.54); see Figure 6.

### 3.2. Sub-Analysis

Several age cutoff points were investigated based on regression analysis. The results showed that patients aged 30 years old who were taking infliximab developed more ADAbs compared to those ≤30 years old [geometric mean (GM) 3.50 vs. 4.28, (0.82, 95% CI; 0.977–0.982) *p* = 0.003], whereas, in patients taking adalimumab there was no statistically significant difference among the level of ADAbs between patients younger and older than 30 years of age [GM 5.89 vs. 5.87, (1.03, 95% CI; 0.982–1.004) *p* = 0.351] (Table 2).

Similarly, a comparison of drug levels among patients aged ≤30 vs. >30 years old (Table 3) showed that patients on infliximab therapy that were older than 30 had lower drug levels compared to patients ≤30 [GM (1.22, 95% CI; 1.10–1.982) *p* < 0.001], whereas in patients taking adalimumab, there was no statistically significant difference in drug level between patients ≤30 compared to patients >30 years old [GM 1.61 vs. 1.46, (1.10, 95% CI; 0.94–1.875) *p* = 0.240]. On the other hand, when investigating drug levels among patients aged ≤18 vs. >18 years old, there was no difference between the infliximab levels in patients younger than 18 compared to patients older than 18 [GM (1.12, 95% CI; 0.89–1.23) *p* = 0.342]. And similar results were observed for adalimumab levels in the same-age comparison [GM (0.94, 95% CI; 0.78–1.01) *p* = 0.246]. There were not enough patients above the age of 65 to perform an analysis of this population.

A total of 359 patients were included in the sub-analysis based on recipients of combination therapy; of those, 128 patients were >30 years of age. There were only nine patients >65 years of age that received combination therapy. When looking at patients who were receiving anti-TNF combination therapy, there was no significant difference in ADAbs levels among patients aged ≤30 vs. >30 years old. Specifically, in patients taking infliximab combination therapy, the ratio of the geometric mean was 1.07 (95% CI, 0.98–1.02, *p* = 0.423). Similarly, in patients taking adalimumab combination therapy, the ratio of the geometric mean for ADAbs was 1.10 (95% CI, 0.82–1.98, *p* = 0.213).

Similarly, in patients receiving anti-TNF combination therapy, there was no significant difference in serum drug levels among patients aged ≤30 vs. >30 years old. Specifically, in patients taking infliximab combination therapy, the ratio of the geometric mean was 0.99 (95% CI, 0.45–1.62, *p* = 0.070). Similarly, in patients taking adalimumab combination therapy, the ratio of the geometric mean for ADAbs was 1.11 (95% CI, 0.559–1.538, *p* = 0.125).

In an additional analysis, the effects of BMI (≥30 kg/m^2^ vs. <30 kg/m^2^), serum albumin [≥40 g/L (normal) vs. <40 g/L (abnormal)], and smoking (smoker vs. non-smoker) on average levels of ADAbs were investigated. The analysis showed no statistically significant difference in average levels of ADAbs between patients receiving infliximab with a BMI of ≥30 kg/m^2^ compared to patients with <30 kg/m^2^ (12.4 vs. 18.2 AU/mL, *p* = 0.125). Similar findings were observed across the two categories of albumin, and there was no statistically significant difference (11.6 vs. 20.4 AU/mL, *p* = 0.148). Finally, smoking status did not affect the average levels of ADAbs (10.1 vs. 19.5 AU/mL, *p* = 0.181). Similarly, in patients receiving adalimumab, these factors did not affect the levels of ADAbs.

## 4. Discussion

In this current retrospective, nationwide study, we have found that as age increases, the infliximab serum level decreases and the amount of antidrug antibodies (ADAbs) increases. However, no correlation between age and adalimumab serum or ADAbs levels was observed.

A recent study found that older adults receiving infliximab were significantly more likely to develop ADAbs compared with younger adults with IBD. In that study, data were extracted from the laboratory information system without any personal or clinical patient information. Furthermore, immunomodulator use was not accounted for, though this can affect the development of ADAbs. Additionally, concentrations of infliximab and its ADAbs were determined using drug-tolerant homogeneous mobility shift assays. Interestingly, individuals ≥60 years of age developed more ADAbs in response to infliximab compared to individuals <60 years of age (*p* < 0.01). Researchers have attributed this to the fact that older individuals were also less likely to receive escalated infliximab doses compared with younger individuals [19].

Studies have found that the presence of ADAbs is correlated with a lower drug concentration [20,21,22]. Additionally, the connection between drug levels with clinical response and remission has been demonstrated in multiple studies [23,24,25]. These studies showed that if serum drug levels are adequate, the rate of remission in both CD and UC and with different drugs is higher.

A prospective UK-wide observational study was conducted in a cohort of 1610 patients (955 treated with infliximab and 655 treated with adalimumab). All patients were anti-TNF-naive with active luminal CD. The study found that obesity, smoking, low albumin concentrations, higher baseline markers of disease activity, and development of immunogenicity were all associated with low drug concentrations and anti-TNF failure [4].

In the present study, we have also found that patients receiving infliximab who are more than 30 years of age develop more ADAbs; subsequently, serum infliximab levels were lower in this population of patients. This could be due to older adults being less likely to receive higher doses of infliximab or combination therapy, despite the fact that these strategies can be safely used in older adults [26].

An observational study was conducted in patients with inflammatory joint diseases to investigate the associations between serum adalimumab and risk factors for developing ADAbs. It was a registry-based study and 340 patients with different with inflammatory joint diseases were included. Similarly to our findings, the authors did not find a statistically significant association between age and formation of ADAbs in patients taking adalimumab [27].

In another study conducted between 2019 and 2021, a total of 430 patients receiving infliximab or adalimumab for IBD or rheumatoid arthritis were recruited in the therapeutic drug-monitoring (TDM) unit.

Antidrug antibodies were detected in 31.5% of serum samples. Notably, the formation of ADAbs against infliximab and adalimumab was significantly higher in the pediatric age group compared to the adult age group, and this difference persisted throughout the study period. Furthermore, pediatric sera exhibited an earlier onset of ADAbs compared to adult sera. Interestingly, pediatric female sera demonstrated the most rapid development of ADAbs [10].

It is important to note that ADAbs can be categorized into two types based on their impact on drug level. The first type are non-neutralizing ADAbs, which do not interfere with the function of the drug, and the second type are neutralizing ADAbs, which inhibit the pharmacological activity of the drug. A drug inducing neutralizing ADAbs against anti-TNFs can have a negative impact on the course of treatment [28]. Some tests specifically determine the type of antibodies, and this would be useful from a clinical perspective. However, these tests are not widely available [29].

This study has several clinical implications. Identifying factors that are associated with treatment failure will aid in facilitating early dose optimization, optimal treatment options, and direct monitoring. Furthermore, the use of strategies to mitigate the development of ADAbs in older patients, such as the use of immunomodulators, higher infliximab dosing, and possibly proactive therapeutic drug monitoring, will allow biologics to be used in a safer and more effective manner. Therefore, continued education regarding the high risk of ADAbs development in older patients receiving infliximab needs to be addressed.

Our study has several strengths. To our knowledge, this is the first study that investigated age as a cause of anti-TNF failure in a large real-world setting. Furthermore, it is a well-designed study with strict inclusion and exclusion criteria to minimize bias and confounders. It was also conducted across multiple hospitals, which involved data from diverse patient populations over a more than 5-year period. This study also addresses a gap in the literature and supports future research in this area.

Reduced statistical power, loss of information, variability in the data, and potential confounding are the major issues associated with the categorization of continuous variables in clinical research. Given these hindrances, we did not analyze ADAbs and drug concentrations as dichotomous/categorical variables (e.g., normal/low vs. abnormal/high). Alternatively, we have analyzed the outcome variables (i.e., ADAbs and serum drug concentrations) as continuous variables.

This study has some limitations. The retrospective nature of this study can introduce some unintentional confounding effects; for example, patients receiving monotherapy could have been prescribed combination therapy previously, only to discontinue the treatment plan due to an adequate serum drug concentration and low ADAbs. Moreover, TDM tests were performed reactively or proactively depending on the physician’s clinical judgment, and the effect of TDM type on our outcome was not assessed.

## 5. Conclusions

Patients older than 30 years of age receiving infliximab monotherapy have higher ADAbs and lower serum drug concentrations than younger patients. There was no difference in antidrug antibody and serum drug concentrations among patients receiving infliximab combination therapy or adalimumab monotherapy. Therefore, a number of factors should be carefully considered when starting infliximab therapy in older adults, such as early use of higher dosing, immunomodulator combination therapy, or proactive drug monitoring to minimize the likelihood treatment failure.

## Figures and Tables

**Table 1:** Characteristics of total study sample and subsample.

Variable	Total Study Sample (*n* = 1093)	Subsample * (*n* = 359)
Age (years)		
Median (interquartile range)	26.0 (19.0–36.0)	25.0 (19.0–39.0)
Age group, *n* (%)		
≤30	664 (60.7)	231 (64.3)
>30	429 (39.3)	128 (35.6)
Sex, *n* (%)		
Male	567 (51.9)	187 (52.1)
Female	526 (48.1)	172 (47.9)
Ethnicity, *n* (%)		
Middle East	1007 (92)	330 (91.9)
Others	86 (7.9)	29 (8.1)
Median body mass index (BMI), median (IQR)	23.1 (22.0–24.2)	23.2 (23.0–24.0)
Ulcerative colitis (UC), *n* (%)	486 (44.5)	161 (44.8)
E1: ulcerative proctitis	50 (10)	16 (10)
E2: left-sided UC	147 (30)	48 (30)
E3: Extensive colitis	289 (60)	97 (60)
Partial Mayo score	1.4	1.4
Crohn’s disease (CD), *n* (%)	607 (55.5)	198 (55.2)
L1: ileal CD	280 (46)	89 (45)
L2: colonic CD	55 (9)	18 (9)
L3: ileocolonic CD	242 (40)	79 (40)
L4: upper gastrointestinal CD	30 (5)	12 (6)
B1: non-stricturing, non-penetrating CD	285 (47)	91(46)
B2: stricturing CD	158 (26)	49 (25)
B3: penetrating CD	164 (27)	58 (29)
Harvey–Bradshaw Index (HBI)	2.2	2.3
Median infliximab therapy duration (years), median (IQR)	4.2 (4.0–4.2)	4.3 (4.0–4.4)
Median adalimumab therapy duration (years), median (IQR)	4.6 (4.0–4.6)	4.5 (4.0–4.6)
Anti-TNF drug, *n* (%)		
Infliximab	461(42.2%)	147(40.9%)
Adalimumab	632 (57.8%)	212 (59.1%)
Infliximab serum concentration, (µg/mL)		
Median (IQR)	5.1 (3.8–5.3)	4.6 (3.0–5.3)
Geometric mean (95% CI)	3.3 (2.7–4.1)	2.3 (1.6–3.3)
Adalimumab serum concentration, (ug/mL)		
Median (IQR)	8.9 (8.0–10.2)	8.3 (7.9–9.6)
Geometric mean (95% CI)	8.4 (7.4–9.5)	8.4 (7.0–10.3)
Anti-infliximab antibody serum levels, (AU/mL)		
Median (IQR)	26.9 (26.1–32.2)	27.5 (20.4–30.6)
Geometric mean (95% CI)	29.0 (24.9–33.8)	25.0 (18.8–33.2)
Anti-adalimumab antibody serum levels, (AU/mL)		
Median (IQR)	10.4 (9.1–13.9)	10.9 (9.0–13.2)
Geometric mean (95% CI)	12.2 (10.9–13.7)	11.0 (9.2–13.1)
Active inflammation *n* (%)		
Yes	125 (11)	43 (12)
No	965 (88)	314 (87)
Missing, (*n*)	3	2
Inflammatory markers (mean value)		
C-reactive protein, mg/L	7.8	7.7
Albumin, g/L	38.9	38.8
Fecal calprotectin, ug/g	130.4	130.5

CI—confidence interval; NA—data not available—IQR, interquartile range. * Subsample includes patients with information on immunomodulators (combination therapy). * The subsample is part of the whole cohort.

**Table 2:** Table showing geometric mean of antidrug antibody levels in patients taking infliximab and adalimumab stratified by age (below or above 30 years of age).

Antidrug Antibody Level (AU/mL) Comparison						
Drug	*n* (≤30)	Geometric Mean	*n* (>30)	Geometric Mean	Ratio of Geometric Means * (95% CI) (≤30 vs. >30)	*p*-Value
Total Sample						
IFX	314	3.50	147	4.28	0.82 (0.977–0.982)	0.003
ADL	350	5.89	282	5.87	1.003 (0.982–1.004)	0.351
Anti-TNF Combination Therapy						
IFX+	105	5.46	42	5.08	1.07 (0.98–1.02)	0.423
ADL+	126	3.72	86	3.36	1.10 (0.82–1.98)	0.231

* Adjusted for sex, age at assessment, active inflammation, ADL—Adalimumab, IFX—Infliximab, and patients with immunomodulators.

**Table 3:** Table showing geometric mean of drug levels in patients taking infliximab and adalimumab stratified by age (below or above 30 years of age).

Drug Level (µg/mL) Comparison						
Drug	*n* (≤30)	Geometric Mean	*n* (>30)	Geometric Mean	Ratio of Geometric Means * (95% CI) (<30 vs. >30)	*p*-Value
Total Sample						
IFX	314	4.28	147	3.50	1.22 (1.10–1.982)	<0.001
ADL	350	1.61	282	1.46	1.10 (0.94–1.875)	0.240
Anti-TNF Combination Therapy						
IFX+	105	1.97	42	1.98	0.99 (0.45–1.62)	0.070
ADL+	126	2.83	86	2.54	1.11 (0.559–1.538)	0.125

* Adjusted for sex, age at assessment, active inflammation, ADL—Adalimumab, IFX—Infliximab, and patients with immunomodulators.

## Data Availability

Data are available on request from the corresponding author due to local legal and ethical restrictions.

## Abbreviations

The following abbreviations are used in this manuscript:

ADAbs	Antidrug antibodies.
Anti-TNF	Tumor necrosis factor antagonists.
IBD	Inflammatory bowel disease.
CD	Crohn’s disease.
UC	Ulcerative colitis
